# Integrating Youth Perspectives: Adopting a Human Rights and Public Health Approach to Climate Action

**DOI:** 10.3390/ijerph19084840

**Published:** 2022-04-15

**Authors:** Giulia Gasparri, Yassen Tcholakov, Sophie Gepp, Asia Guerreschi, Damilola Ayowole, Élitz-Doris Okwudili, Euphemia Uwandu, Rodrigo Sanchez Iturregui, Saad Amer, Simon Beaudoin, Mayumi Sato

**Affiliations:** 1 Department of Land Economy, University of Cambridge, Cambridge CB3 9EP, UK; 2 Department of Epidemiology Biostatistics and Occupational Health, Faculty of Medicine and Health Sciences, McGill University, Montreal, QC H3A 1G1, Canada; yassen.tcholakov@mcgill.ca; 3 Charité – Universitätsmedizin Berlin, Corporate Member of Freie Universität Berlin and Humboldt-Universität zu Berlin, 10117 Berlin, Germany; geppsophie@gmail.com; 4 Department of Humanities, University of Ferrara, 44121 Ferrara, Italy; asia.guerreschi@gmail.com; 5 Department of Medicine and Surgery, Faculty of Clinical Sciences, Obafemi Awolowo University, Ife 220101, Nigeria; damilolaayowole90@gmail.com; 6 Economic Community of West African States (ECOWAS) Parliament, Abuja 900103, Nigeria; elitzdorisokwudili@gmail.com; 7 Concerned Group for Environment, Population and Development in Nigeria (N-COGEP-D), Owerri 460242, Nigeria; sharpay136@gmail.com; 8 School of Medicine, Faculty of Human Medicine, Antenor Orrego Private University, Trujillo 13009, Peru; rsancheziturregui@gmail.com; 9 Plus1Vote, New York, NY 33301, USA; saadamer@plus1campaign.org; 10 Department of Politics and International Studies, Faculty of Human, Social and Political Science, University of Cambridge, Cambridge CB3 9DP, UK; sb2421@cam.ac.uk; 11 Samuel Centre for Social Connectedness, Samuel Belonging Lab, Toronto, ON M4W 2C1, Canada; msato@scscglobal.org

**Keywords:** climate change, public health, human rights, co-benefits, intersectionality, youth engagement

## Abstract

Climate change is a multidimensional issue that affects all aspects of society, including public health and human rights. Climate change is already severely impacting people’s health and threatening people’s guaranteed fundamental rights, including those to life, health, self-determination, and education, among others. Across geographical regions, population groups and communities who are already marginalized due to age, gender, ethnicity, income, and other socioeconomic factors, are those who are disproportionately affected by climate impacts despite having contributed the least to global emissions. Although scholars have been calling for a human rights-based approach and a health perspective to climate action, the literature looking at this multidisciplinary intersection is still nascent, and governments have yet to implement such intersectoral policies. This commentary begins to reflect on the relationship between climate change, human rights, and public health from the perspective of young people engaged in climate action and discourse at the national and international levels. It presents a way forward on what we, as youth climate advocates and researchers, believe is a priority to bring intersectoral integration of human rights and public health approaches to climate change to fruition. First, scholars and practitioners should examine and support youth-led climate interventions that tackle human rights and public health violations incurred by the climate crisis. Second, participatory approaches to climate change must be designed by working synergistically with climate-vulnerable groups, including children and young people, practitioners and scholars in public health and human rights sectors to holistically address the social, health, and environmental impacts of the climate crisis and root causes of injustice. Finally, we recommend more holistic data collection to better inform evidence-based climate policies that operationalize human rights and public health co-benefits.

## 1. Introduction

The latest Physical Science and Impacts, Adaptation and Vulnerability reports of the Intergovernmental Panel on Climate Change (IPCC) foreshadow cataclysmic visions of humanity’s future [1,2]. The proclamations by the United Nations Secretary-General António Guterres of a “code red for humanity” indicate that climate change has seeped its way into every facet of social, cultural, political, and economic life [3]. Recent climatic events have seen rising temperatures across North America and Europe, rampant floods in China and Germany, severe desertification in Southern Africa, and wildfires in Greece, North America, Australia, and Russia, reaffirming that these events are no longer aberrations [4]. Rather, they indicate pervasive and intensifying changes that will continue to exacerbate human rights violations, jeopardize the health of climate-vulnerable and frontline communities, threaten food, land, and water security, and reverse decades of advancements made in social, gender, and political justice [2,4]. Current delivery on mitigation policies puts us on track for at least a 2.7 °C warming by 2100, rather than the global target of 1.5 °C, and G20 countries, who account for nearly 80% of global greenhouse gas (GHG) emissions, are expected to fall short of their Nationally Determined Contributions (NDCs) [4,5].

Between 3.3 and 3.6 billion people are estimated to live in conditions of high vulnerability to climate change [2]. People’s vulnerability to climate change is highly dependent on the contextual challenges of certain regions and population groups, due to continuing historical patterns of inequity linked to gender, age, ethnicity, low income, and colonialism [2]. According to the IPCC, “global hotspots of high human vulnerability are found in particular in West-, Central- and East Africa, South Asia, Central and South America, Small Island Developing States and the Arctic”, as well as in locations of high poverty and limited access to basic services and resources [2]. Evidence shows that “between 2010–2020, human mortality from floods, droughts, and storms was 15 times higher in highly vulnerable regions, compared to regions with very low vulnerability” [2]. These regions are also where the largest absolute number of people displaced by climate change can be found [6]. Studies show that poorer households are often more likely to live in locations highly exposed to extreme heat and air pollution, and are more likely to be forced to migrate [6].

Furthermore, climate vulnerability is highly gendered and depends on socioeconomic contexts, where the impacts of and burdens of responding to climate change affect communities who are often suffering from mutually informing struggles for human rights and health equity. For example, women living in rural communities in the Global South who subsume most household duties and endure longer distances outdoors for the collection of water and food due to climate-induced food and water insecurity, face increased exposure to overheating, air and water contamination, and are subject to greater risk of gender-based violence during their commute [7]. Evidence shows that pregnant women and newborns are significantly threatened by heatwaves and air pollution, causing higher chances of miscarriages, preterm birth, and poorer neonatal outcomes, as well as food insecurity, leading to undernutrition among pregnant women [8]. This increases the likelihood of children suffering from low birth weight, one of the main risk factors for infant mortality and morbidity [9]. UNICEF estimates that over 1 billion children, half of today’s children, live in countries at extremely high risk of climate change, which puts their survival in jeopardy, specifically by undernutrition, air pollution, and diseases [10]. This severely affects children’s mortality and morbidity, with high expectations of rising cases of diarrhea and pneumonia [10]. Even under current emission reduction pledges scenarios, a person born in 2020 is going to experience a two- to sevenfold increase in weather events, especially heatwaves, compared to those born in the 1960s, which will have profound impacts on their development and well-being [11].

Given that the Universal Declaration of Human Rights enforces that all individuals are entitled to the same rights and freedoms enshrined in the Declaration, regardless of national origin, gender, age, and race, and given that climate-induced changes are disproportionately targeting the health and livelihoods of already marginalized groups on a systemic level, who are least responsible for GHG emissions, it becomes clear that climate change threatens their ability to exercise their rights to a free, healthy, and dignified life [12]. Therefore, as the IPCC states, climate-resilient development requires policies and interventions that aim to decrease exposure, vulnerability, and societal inequity, while simultaneously addressing effective mitigation and adaptation in order to reduce climate change’s impacts on health, well-being, and human livelihoods [6].

This commentary reflects on the relationship between climate change, human rights, and public health from the perspective of youth engaged in climate action and discourse at the national and international levels. It argues that a human rights and public health lens to climate change action can more effectively ensure climate-resilient development and reduce the vulnerabilities of the most affected and impacted groups. Such an approach could not only help strengthen communities’ resilience but also foster multisectoral mitigation and adaptation measures that address multiple pillars of the sustainable development goals. Specifically, we explore the role of youth-led intersectional interventions in ensuring equitable engagement in climate decision making and climate-just solutions, which simultaneously address the human rights, health, and well-being of the most affected and vulnerable groups aiming to enhance their resilience. In this commentary, we draw on the definition of intersectionality rooted in the work of Kimberlé Crenshaw as a critical tool to identify the most marginalized communities within a particular spatial and cultural context, and offer insights on how these identities’ rights can be upheld [13]. We then present a way forward on what we, as youth climate advocates and researchers, believe is a priority to foster intersectoral human rights and public health approaches to climate change. 

## 2. Recognizing the Multidimensional Impacts of the Climate Crisis

The climate crisis has been increasingly acknowledged by the scholarly and health community as one of the greatest threats to public health and human rights [14,15,16,17,18,19,20]. There is ample research showing how climate change is already affecting people’s physical and psychological health, through both direct exposure to extreme weather events, such as heatwaves, storms, and floods, and indirect disruption to food systems, water security, and health care services [6,14,15]. Worldwide temperature and humidity increase is causing a rise in cases of vector-borne diseases (such as dengue, chikungunya virus, Lyme disease), diarrheal diseases, and foodborne diseases [6]. Climate-associated health risks are expected to further burden climate-vulnerable communities, in particular people from low-income backgrounds, racialized groups, Indigenous people, women, children, older adults, and those already suffering from chronic disease [15,21,22]. Variabilities in temperatures, especially heatwaves, and exposure to climate hazards particularly impact maternal and neonatal health, including higher rates of preterm birth, low birthweight, stillbirth, and neonatal stress, leading to adverse child health in the long term [6]. Vector-borne diseases are associated with sociodemographic factors, including economic status, housing quality, and susceptibility, which puts that same population at greater risk [6]. Under high emission scenarios, it is estimated that 48,000 more children under 15 years of age could die of diarrhea in 2030 [6]. Cases of asthma, the most common chronic disease among children and adolescents worldwide, are increasing due to rising temperatures [23,24]. Extreme weather events can also limit young girls’ access to sexual and reproductive health services, leading to an increase in unwanted pregnancies, complications and death during childbirth, and sexually transmitted infections (STIs) [8]. Consequently, the World Health Organization has stressed that “the climate crisis threatens to undo the last fifty years of progress in development, global health, and poverty reduction, and to further widen existing health inequalities both between and within populations” [14]. Projections show that between 2030 and 2050 there will be an additional 250,000 deaths per year due to climate change-induced heat, undernutrition, malaria, and diarrheal diseases, with more than half of this mortality being in Africa [6].

Moreover, climate change-induced weather events also affect mental health, particularly for low-income populations, leading to post-traumatic stress disorder (PTSD), anxiety, substance abuse, and depression [25,26,27]. Children, adolescents, and young people, especially girls, carry a substantive burden, as they are more susceptible to experiencing PTSD, eco-anxiety, and depression with lifelong impacts following a climate-related disaster or in response to political inaction to climate change [6,28,29].

Furthermore, climate change is disrupting people’s livelihoods by affecting agricultural production, contaminating water and air, rendering lands uninhabitable, and forcing migration [30]. Low-income countries, whose economies are more agriculturally dependent, and already marginalized groups, face the highest climate vulnerability, further exacerbating global social injustice [2]. To exemplify, compared to adults, children have higher chances of experiencing malnutrition, which already affects one in three children worldwide, linked to climate-induced water and food shortage [22]. Estimates show that the number of deaths of children under five caused by undernutrition could increase by 1 million by 2030 due to climate change [6]. Indigenous people and children, who often live on low-elevation deltas, are more susceptible to facing high sea level rise and flooding, and are seeing their cultural relationship to nature severely threatened by deforestation and land degradation [22].

Herein, it can be noted that the fundamental rights enshrined in the Universal Declaration of Human Rights, as well as in the Convention on the Rights of the Child, including the right to life, health, food, and a range of cultural rights, are in jeopardy [12,17,31,32,33]. The Office of the United Nations High Commissioner for Human Rights (OHCHR) has increasingly recognized that climate change is a human rights issue, given the profound threats posed by extreme weather events, sea level rise, climate-induced vector-borne diseases, and food and water insecurity to the right to life and health of many [34]. The extraction of fossil fuels, the driving cause of climate change, is also often connected to human rights violations, due to frequent expropriation of Indigenous people’s land or uncontrolled pollution [35]. The inequity of these impacts is undeniable. Climate resilience is not equally distributed among the world, with specific regions such as coasts, small islands, deserts, mountains, and polar regions being most affected, and already vulnerable and marginalized communities owing to age, gender, income, ethnicity and geography bearing the greatest burden of impacts, despite having contributed least to climate change [2,12]. Specifically, in the case of children, climate change undermines the effective enjoyment of children’s rights enshrined in the Convention on the Rights of the Child [10]. Due to the interconnected nature of rights, the realization or violation of one right often depends on other rights; therefore, the Committee on the Rights of the Child has stated that climate change is one of the biggest threats to children rights including: the right to survival and development (article 6), the right to health (article 24), the right to adequate standard of living (article 27), and the right to education (article 28), among many others [10]. For example, extreme weather events such as droughts and floods as well as air pollution are affecting children’s access to school due to destruction of school infrastructure, preventive school closures, injuries of parents and teachers resulting in absenteeism [36]. Floods in Cambodia during 2013–2014 saw 155 schools closed for up to nine weeks, 40,000 textbooks destroyed, more than 450,000 students unable to access schools, resulting in an increase in dropout rates and grade repetitions [36]. Even within vulnerable population groups, the impacts are not equal across the world. Children who live in fragile or humanitarian settings are also those who are most vulnerable to climate change, often resulting in the highest levels of displacement and consequently high risk of further human rights violations such as exploitation and violence, family separation resulting in trauma, and lack of access to education [10].

Therefore, it is undeniable that climate change profoundly impacts universal human rights both directly and indirectly [34]. To reduce the negative impacts and build climate resilience among the most affected, a rights-based approach is paramount [12]. Similarly, throughout the years, the Conference of the Parties to the United Nations Framework Convention on Climate Change (UNFCCC) has increasingly recognized that “[p]arties should, in all climate change-related actions, fully respect human rights” and has emphasized that climate interventions must be undergirded by human rights principles [22,37].

## 3. Adopting Intersectoral Frameworks to Address the Climate Crisis

The increased recognition that climate change is jeopardizing human rights, including the universal right to health, especially those of marginalized communities has brought scholars and the OHCHR to call for the adoption of a human rights-based approach to address climate change (HRBA) [37,38,39,40,41]. A HRBA responds to the dimensions of climate justice and the need to develop mitigation and adaptation policies, as well as loss and damage frameworks, that put those who are disproportionately impacted by climate change at the center [41]. When implemented through a human rights lens, mitigation and adaptation measures are designed for and with the participation of the most vulnerable, following the principles of human rights law [36]. As illustrated by the recent reports of the IPCC, a HRBA can help reduce structural vulnerabilities to climate change by addressing structural inequities linked to age, gender, ethnicity, disability, location, and income through providing the most marginalized and affected with capacity building, meaningful participation and inclusion, and targeted resources, financing, and policies to better adapt and build resilience [2]. Additionally, when using a HRBA, the human rights legal framework can assist right holders to hold governments accountable if their climate policies result in, or normalize, human rights violations against individual citizens and communities, including to their right to health [39,40]. For example, in February 2020, German youth filed a plaintiff against Germany’s Federal Climate Protection Act claiming it was violating human rights, specifically the right to life and physical integrity, as well as the rights of future generations, by not requiring sufficient short- and medium-term emission reductions [42]. The Court ruled in favor of the complainants, ordering the legislature to establish further emission reduction targets as “subsequent generations [would otherwise be forced to] a drastic reduction burden and expose their lives to serious losses of freedom” [42]. In another case, the Colombian Constitutional Court ruled in favor of Indigenous communities claiming that the pollution of the Atrato river was a violation of their rights to life, health, water, food security, healthy environment, culture, and land property and requested the government to assess all mining and energy policies against the effects of climate change in order to protect human rights [43].

Similarly, to the call for a HRBA to climate change action, scholars argue that integrating climate mitigation and adaptation interventions that directly improve people’s health, not only alleviates the impacts of climate change on climate-vulnerable communities, but also reduces pressure on health systems and provides cost-effective health promoting measures [13,44,45,46]. Universal access to primary health care, including mental health, increased investments in health systems strengthening, and climate-resilient health systems, is one of the means to increase the climate resilience of populations and ensure adaptation [6]. Similarly, stronger commitment and implementation of vector control management approaches, disease surveillance, early warning systems, and equitable vaccine production and distribution can enhance prevention and preparedness to future vector-borne diseases, also drawing from the lessons of the COVID-19 pandemic [6]. The recent IPCC report finds that the co-benefits to health and well-being, such as avoided number of hospitalizations, morbidity, and premature deaths, exceed the financial costs of mitigation measures [6]. To exemplify, reducing air pollution from the transport sector contributes to climate mitigation and improves air quality, therefore reducing the number of premature deaths caused by air pollution worldwide (a number that is estimated to be around 7 million yearly) [47]. The IPCC estimates that the financial returns in terms of health benefits of improved air quality are greater than the costs of reaching the Paris Agreement goals [6]. This also connects with justice and equity, as in many parts of the world, poor and marginalized communities are more affected by air pollution [48]. The health community is therefore increasingly pushing for governments to account for these health benefits of climate action, as this can lead to more incentives for just, ambitious, and cost-effective climate action [14,20,44,46,49].

Yet, despite the increased evidence of the health consequences of climate change, as well as the threats to human rights, current adaptation and mitigation measures rarely safeguard communities and promote social justice-oriented solutions that alleviate existing inequities faced by climate-vulnerable populations [22]. The failure of Global North countries to fulfill their duty of compensating vulnerable countries suffering loss and damage for their disproportionate emissions continues to show the reluctance of some governments to adopt a HRBA [50]. During the 26th United Nations Climate Change Conference (COP26) negotiations, many activists have called out countries wanting to erase explicit references to climate-induced health and human rights violations [50]. We join activists claiming that global leaders seem to have forgotten the world’s most affected communities, especially young people’s needs and future generations’ priorities [50]. Children are systematically overlooked in climate policies, with only 42% of NDCs having reference to children or youth [10]. Additionally, according to UNICEF, only three countries refer specifically to children’s rights and only five have specific reference to human rights with regards to future generations and intergenerational equity [10]. Nearly 23% do not mention children or youth, nor their well-being, such as education [10]. UNICEF also registers a lack of disaster risk-reduction strategies specifically targeted at children [10]. While literature has attributed disproportionate impacts of climate change to women, gender-responsive measures, including reference to aspects of sexual and reproductive health and rights, and its intersection with human rights are not extensively reflected in countries’ NDCs [8]. Moreover, in NDCs commitments, there is a lack of explicit reference to health co-benefits, and the health impacts of climate change are rarely linked to quantified evidence and policies [45]. For example, climate laws and adaptation plans in Brazil and Colombia lack explicit reference to a HRBA and weakly integrate the right to health, failing to ensure that public healthcare systems are prepared to respond to the health needs of the most affected communities [51].

Policies that include co-benefits have to be designed such that equity and justice are taken into account to ensure that inequities are not exacerbated. As illustrated above, those whose health is at risk are often the same climate-vulnerable communities who are most likely to be subject to human rights violations; we argue that adopting a HRBA and a health lens to climate action can thus lead to more just and cost-effective solutions aiming at addressing the climate resilience of the most impacted and vulnerable groups among and within societies, including children and young people. As illustrated by the IPCC, adopting such an intersectoral approach, which recognizes the systemic discrimination that vulnerable communities face, due to their age, gender, ethnicity, disability, income, and socioeconomic status among others, with a human rights and health lens to tackle the climate crisis, will lead to more ambitious, comprehensive, and cost-effective interventions to increase climate resilience and action [2].

## 4. Young People at the Forefront

Throughout history, young people have often been at the forefront of social and political movements, mobilizing communities to demand accountability and change, pressuring decision makers to broaden their priorities beyond economic interests, and voicing concerns of those who are systematically overlooked [52]. Given the disproportionate impact that young generations and future ones will be forced to bear, across the globe, young people possess a voice with significant moral authority and have been called “new ambassadors for scientific consensus and climate mitigation” [53]. They are demonstrating their understanding that public health, human rights, and climate change are not only interlinked, but inextricably tied to dimensions of age, gender, race, sexual orientation, and income, among others. Today’s cohort of children and young people is the largest the world has ever known, accounting for nearly 24% of the global population [54]. As they will inherit the world, youth voices and priorities must therefore be reflected, despite many not yet having the right to vote.

The Global Youth Statement, presented by the UNFCCC Children and Youth constituency (YOUNGO) at COP26, calls governments to adopt a holistic and multifaceted approach to climate action that “respect[s], protect[s], fulfil[s], and promote[s] all human rights … address[es] the disproportionate effects of the climate crisis borne by under-privileged or marginalized communities” and acknowledges and responds to “the dual crisis of a global health and climate emergency” [55]. In particular, young people have mobilized globally, calling for a shift from short-term climate solutions, drawing attention to a radical reimagining of how institutions should better care for the health and rights of marginalized communities [56]. This has led to youth developing campaigns calling for US universities to divest from fossil fuels that aggravate respiratory conditions, to attempting to unload coal from machineries in Indonesia, fighting against fossil fuels’ health and environmental consequences [56]. In particular, marginalized youth have been active agents on the frontlines, drawing attention to the ways in which fossil fuel extraction, deforestation, and coal mining, harm individual and community health, and disrupt harmonious relationships between people and nature [56].

In addition to their advocacy efforts, young people have also been applying a human rights and public health lens in their climate litigation efforts against national governments. South African youth have brought forth litigation to uphold their Constitutional rights to live in an environment that is not harmful to their health and one that protects future generations from pollution and ecological degradation, in light of heatwaves, droughts, poor sanitation, and water access, which have prevented adolescents to access and attend school [57]. In 2019, 16 children filed a claim against the governments of Argentina, Brazil, France, Germany, and Turkey for failing to rapidly reduce their greenhouse gas emissions, and therefore violating children’s rights to life, health, and the prioritization of the child’s best interest under the United Nations Convention on the Rights of the Child [58]. In Colombia, the Supreme Court of Justice ruled in favor of 25 children and youth who argued that the deforestation of the Amazon rainforest is threatening their right to a healthy environment, life, and risking future generations [59]. With this historic ruling, the government was ordered to create an “intergenerational pact for the life of the Colombian Amazon” with the engagement of the plaintiffs, and municipalities were ordered to develop action plans to reduce deforestation [59]. Six Portuguese youth have also filed a lawsuit against 33 European countries with the European Court of Human Rights (ECHR) [60]. With this first climate lawsuit filed with the ECHR, youth applicants contended that climate change is threatening their right to life, private and family lives, and right not to be discriminated against, among others. The lawsuit stressed the severe impact climate change is having on their physical and mental health, requiring European governments to implement more ambitious emission reductions and dramatically reduce fossil fuel exports [60]. Youth-led lawsuits and the mobilization of grassroots climate activism have used arguments for the security of good health and rights to a healthy and sustainable life as the entry point for their action. These cases demonstrate how youth have vouched for the integration of public health and human rights interventions to climate change through legal and policy channels, to create policy pathways that are justice oriented and centered most around communities on the margins.

From the organization of protests to the mainstreaming of public awareness around the climate crisis through social media, engaging in community action, and filing lawsuits, young people have solidified themselves as pioneers in applying a human rights approach to climate change while highlighting the health and social impacts of climate change [61]. These examples demonstrate some of the ways in which young people are attempting change within their own communities, to ensure their rights are upheld, and inspiring novel approaches to climate policies. This can lay the foundations to further pursue intersectional approaches to addressing the climate crisis.

## 5. A Way Forward

While there is growing scholarly attention to the link between climate change, human rights, and public health, as well as the potential application of a public health-based and a human rights-based approach to existing climate pledges and policies, governments still fail to fully recognize and implement these potential co-benefits.

Youth have underscored that an intersectional public health and human rights approach to climate change can lead to more effective, just, and cost-efficient climate action. As youth climate advocates, and researchers with direct experience and work in the climate space, we are well positioned to call upon governments to take and apply a holistic lens that integrates health and human rights perspectives in climate solutions. We offer three recommendations and strategies on how to move forward with climate solutions through holistic approaches, and provide new insights on how to pursue a resilient, equitable, and socially just future.

### 5.1. Look to and Support the Work of Young People

Acknowledging the intersectional perspective and actions brought forward by youth can help scholars and practitioners understand how climate solutions must fundamentally address the intersection of health, human rights, race, class, gender, geography, and power in determining which groups need to be prioritized. With the urgency to mitigate climate change impacts, young people have recognized the need for imminent action, and have mobilized through community action, legal pursuits, and confrontation with State and corporate emitters. Youth-led climate interventions that have held heavy emitters accountable through legal avenues of recourse and advocacy, provide useful insights for health, climate, and human rights scholars and practitioners to adopt similar approaches to reconcile human rights violations and public health threats incurred by the climate crisis. In particular, the intersection of human rights and health discussed in the sections above reveals how the impacts of climate change can be attenuated by focusing on justice and building the resilience of the most vulnerable, including children and young people. Supporting young people through funding, capacity building, collaborative research projects, employment, and opportunities to meaningfully engage in decision making can foster the pursuit of holistic and integrated approaches to climate action.

### 5.2. Adopt Intersectoral and Inclusive Approaches to Research, Policy Design and Implementation, and Monitoring and Evaluation

Addressing climate change beyond an environmental issue to incorporate human rights and public health intersectional approaches requires governments, civil society, and the private sector to work synergistically with practitioners and scholars in the public health and human rights sectors, as well as marginalized communities in research projects, policy design and implementation, and monitoring and evaluation (M&E) [62]. Including the voices of young people, who will be most impacted in the long term, is paramount. Adaptation and mitigation measures should be designed and implemented with the participation of affected communities to develop integrated and comprehensive responses to challenges [63]. An intersectoral and inclusive governance framework, which aims to encompass the diversity of interests, perspectives, and impacts of climate change at the national, regional, and/or municipal levels, is vital for encouraging ownership, capacity, and consensus around policies, as well as for successful implementation, which can be achieved when every impact of climate change is taken into account [64]. As demonstrated in the case studies of young people mobilizing for climate action through litigation and grassroots mobilization, youth are already driving intersectoral accountability on governments’ inaction on multiple dimensions. Including youth and their knowledge can spur greater evidence-based policymaking, M&E, and more cost-effective measures in addressing the social consequences of the climate crisis [65]. This approach also helps to avoid violating protected rights even more so now that participatory rights are part of both human rights law and environmental law as well many national laws [41].

### 5.3. Ensure Integrated Data Collection to Inform Evidence-Based Policies That Take Advantage of Co-Benefits

As illustrated in Section 2 and Section 3, more data is needed to better encapsulate and capture the interconnected health and human rights facets of the climate crisis in order to design integrated human rights and public health-based approaches to climate policy and implementation. Data collection should include and account for disparities and marginalizing factors that lead to vulnerability, such as age, gender, race, class, income, disability, and geography, in order to assure that policies are not blind or even harmful to these groups but on the contrary promote equity and justice [66]. Having such cross-cutting data at the national level will also guide nation states to include these co-benefits in their NDCs and to develop climate adaptation policies, which take into account the multisectoral impacts of the climate crisis [67]. For instance, such national-level analyses would enable national policymakers to understand what is needed to ensure their healthcare system is prepared and resilient to future climate-induced public health crises, what training is needed for health professionals, and what type of civil society awareness campaigns are required [67]. Frameworks such as the Health in All Policies approach, where the health considerations of communities and people are collaboratively integrated into policymaking across sectors, can ensure that co-benefits in other policy areas, including environmental health and human rights, are realized [68]. Such approaches can be undertaken at all levels and multisectoral synergies can be optimized and inequities avoided. In these processes, engaging youth in data collection can ensure their lived experiences are reflected. Finally, through such integrated data collection, public health, human rights, and climate researchers and practitioners can better inform their advocacy efforts and have more impact as agents for climate action.

## 6. Conclusions

Today, we stand at a critical juncture, with less than a decade left to revert the current scientific trajectory [4]. Recognizing that the least climate-resilient population groups and most affected communities across the globe are already facing catastrophic environmental, health, social, political, and economic losses due to climate change, there is no more time to delay action. Understanding the potential and track record of youth in addressing cross-cutting issues of human rights and health equity within climate discourse and legal action, we need to develop and support youth-led public health and human rights approaches to climate action to address the multidimensional threats of the climate crisis. Based on the work undertaken by young people across the world, this paper has brought forward three recommendations for scholars and practitioners to adopt a more holistic understanding of, and responses to, the climate crisis in order to build a sustainable, equitable, and resilient future. Undertaking such recommendations will help protect people’s health, reduce future health costs and safeguard human rights, ensure that policies take advantage of multisectoral co-benefits, reinforce proactive youth and civil society leadership, recognize the voices and needs of the most affected people and communities, and increase awareness on the multidimensional impacts of climate change among the public to push for greater government accountability and effective action.
